# Development of an intranasal, universal influenza vaccine in an EU-funded public-private partnership: the FLUniversal consortium

**DOI:** 10.3389/fimmu.2025.1568778

**Published:** 2025-05-23

**Authors:** Victor M. Cnossen, Paula C. Leao Moreira, Othmar G. Engelhardt, Jerzy Samolej, Geert H. Groeneveld, Simon P. Jochems, Wesley Huisman, Gabriel K. Pedersen, Katharina Wørzner, Lukas Recek, Giulia Piccini, Claudia M. Trombetta, Amy Aspelund, Alaura Hoag, Manfred Reiter, Carrie Wick, Thomas Muster, Ingrid Maria Catharina Kamerling

**Affiliations:** ^1^ Centre for Human Drug Research, Leiden, Netherlands; ^2^ Medicines and Healthcare Products Regulatory Agency, Potters Bar, United Kingdom; ^3^ Leiden University Center for Infectious Diseases (LU-CID), Leiden University Medical Center, Leiden, Netherlands; ^4^ Department of Internal Medicine - Acute Internal Medicine, Leiden University Medical Center, Leiden, Netherlands; ^5^ Center for Vaccine Research, Statens Serum Institut, Copenhagen, Denmark; ^6^ MediTox S.R.O., Konárovice, Czechia; ^7^ VisMederi S.r.l., Siena, Italy; ^8^ Department of Molecular and Developmental Medicine, University of Siena, Siena, Italy; ^9^ Vivaldi Biosciences, Vienna, Austria

**Keywords:** influenza, vaccine, universal influenza vaccine, FLUniversal, controlled human infection model, public-private partnerships, correlate of protection

## Abstract

Influenza is a significant global health problem, causing disease and hospitalisations in elderly individuals and infants. While updated vaccines are available every year, their effectiveness is moderate at best. FLUniversal is a European Union funded consortium, aiming to develop a universal influenza vaccine by bringing together partners with expertise in different areas of vaccine development. An intranasal live attenuated vaccine, DeltaFLU, will be produced using an innovative platform; preclinical assessment in animal models and clinical studies using a controlled human infection model (CHIM) will be conducted for assessment of safety, immunogenicity and protective efficacy; and finally, comprehensive immunological analysis of blood and nasal mucosa will elucidate vaccine responses and potential new correlates of protection (CoPs). In addition to a universal influenza vaccine, listed as a top priority by the EU, FLUniversal seeks to deliver an enhanced vaccine manufacturing technology that is superior in terms of efficiency, production costs and production speed - especially critical in the face of a potential new pandemic. Moreover, an influenza CHIM with a focus on harmonisation of clinical procedures and assays will be established to generate translatable and reproducible data. Newly generated knowledge on mechanisms of protection, CoPs and new molecular analysis tools may significantly contribute to our knowledge on influenza infection and influenza vaccines. In conclusion, FLUniversal is an innovative and ambitious public-private partnership, aiming to present a new development pathway for influenza vaccines, and maximising impact by bringing together leading partners from academy and industry with a shared purpose of collaboration and innovation.

## Introduction

Influenza is a leading worldwide cause of disease and hospitalisations, affecting elderly and infants most severely (1, 2). Approximately 3 to 5 million severe influenza cases occur each year, and annual deaths are estimated to be up to 600,000 worldwide. Moreover, up to 10% of the general population is affected annually, resulting in lost workdays and a significant economic burden (3). In temperate regions incidence is predominantly seasonal, with outbreaks in the northern hemisphere generally beginning after November, and peaks subsiding before April (4, 5). Currently, several seasonal influenza vaccines are available across a variety of vaccine platforms. However, vaccine effectiveness is moderate at best. Also, vaccine strains need to be updated every year as the haemagglutinin (HA) protein that is the main antigenic target of influenza vaccines, is highly changeable and its evolution is unpredictable (6, 7).

The design for a new influenza vaccine every year is costly and time-consuming. The most widely used production platforms take approximately 6 months; the possibility of a mismatch between available vaccines and circulating strains remains (8). Moreover, clinical evaluation of efficacy is often performed in trials focusing on vaccine immunogenicity to the HA, relying on historic immunological assays predictive of protective efficacy that are possibly inadequate for candidates developed through new platforms (9). Assessing clinical vaccine efficacy in patients is generally complex and expensive: due to the unpredictability of influenza virus circulation, trials often are conducted in multiple geographic locations, and may require up to tens of thousands of participants (10). In addition, these populations are often monitored over multiple influenza seasons, which may further delay vaccine availability and increase the risk of mismatch with newly emerging strains.

Many research teams have been working on the development of universal influenza vaccines, to circumvent the need for yearly updates and to protect against potential future influenza pandemics. This has resulted in the development of new platforms and the design of various vaccine candidates, but major breakthroughs have not been achieved (11). The FLUniversal consortium is developing a vaccine platform consisting of innovative preclinical models, an influenza controlled human infection model (CHIM) to produce results akin to wild-type infection as well as clinical samples, and integrated complex immunological analyses. The platform provides synergy, aids in identifying molecular signatures of protection, and reducing the timeline and risks involved in the clinical development of next-generation vaccines. Using this platform, FLUniversal will develop, manufacture and test an influenza vaccine with the aim of achieving universal or broad cross-protective immunity. Reducing timelines in vaccine design, manufacturing and clinical testing is not only beneficial for the current influenza landscape but also amounts to preparedness for possible future pandemics (12).

CHIMs are innovative clinical trials in which a study population (usually) consisting of healthy volunteers is exposed to the target pathogen and may also be given a vaccine or therapeutic. Conducting a CHIM study facilitates rapid vaccine testing in a relatively small population of healthy volunteers, under safe and controlled circumstances. CHIM studies have been performed for decades; while historically focusing on disease characteristics and immunology, they are increasingly conducted to evaluate new vaccines or therapeutics (13, 14). Clinical endpoints such as protection against virus infection or symptomatic disease are valuable additions to vaccine safety and immunogenicity. In addition, CHIMs can be used to study disease characteristics and immunological endpoints in a thorough and standardised manner (13).

## The FLUniversal consortium

FLUniversal is a public-private partnership funded by the European Union’s Horizon Europe research and innovation programme, which started in June 2023. The consortium consists of 8 partners and brings together leading scientific and academic institutions with partners from industry. A vast amount of experience is shared in the consortium, from all aspects of vaccine development: partners have expertise in vaccine design and manufacturing, (pre-)clinical evaluation, CHIMs, standardisation of assays and immunological assessment of vaccines and influenza infection (15). The partners, goals and methods of FLUniversal are comprehensively outlined at https://www.fluniversal.eu/. In this paper, we outline the different areas of innovation that FLUniversal will contribute to.

## Areas of innovation

### DeltaFLU

FLUniversal aims to advance DeltaFLU, a vaccine containing live replication-deficient influenza strains lacking the NS1 protein. The influenza NS1 protein acts as an interferon antagonist; the attenuated influenza strains lacking NS1 induce an interferon response and lead to durable protection through the induction of memory T and B cells (16–18). DeltaFLU is to be administered intranasally, thus inducing mucosal (IgA and tissue-resident memory mediated) immunity resulting in local protection and prevention of further virus transmission. The vaccine is designed to protect against all influenza virus strains; it targets all viral antigens and induces effective T cell-mediated immunity against conserved internal proteins, and B cell-mediated immunity against the conserved stem structure of the HA, which is typically poorly immunogenic. This broad cross-protection will be achieved using a prime-boost immunisation (PBI) regimen to direct the immune responses to conserved regions of the virus. The PBI regimen is a combination of the DeltaFLU influenza strains lacking NS1, paired with wild-type HA proteins that all have different head regions but homologous stalk regions. This combination is expected to also induce HA stalk directed antibodies, which may confer broad cross-protection against influenza, as the HA stalk region is highly conserved across wild-type influenza strains. Vaccines containing isolated stalk regions generally induce poor immune responses, and are often unstable when combined with other, more immunogenic HA regions. The PBI strategy is a simple yet innovative regimen, with the aim to overcome these challenges in achieving universal protection against influenza. FLUniversal will primarily test the PBI regimen with influenza A group 2 HA proteins (H3 and H7), which have been generated and have met all testing criteria to be manufactured for use in clinical trials in 2025. In parallel, combinations including also influenza A group 1 and B HA proteins are being developed separately from the consortium.

### Vaccine strain production

Most current influenza vaccines consist of inactivated influenza viruses produced in embryonated chicken eggs. Commonly, three or four influenza strains are selected; selected influenza A viruses are usually reassorted with the master strain A/Puerto Rico/8/34. These hybrid viruses are then inoculated into the eggs, from which the virus can be harvested, purified, chemically inactivated or split, and formulated to produce an inactivated vaccine. Licensed live attenuated vaccines, based on influenza virus strains with multiple cold-adapted and other function-modulating point mutations, also are produced on embryonated chicken eggs. The time between strain selection and vaccine distribution is approximately 6 months. This time gap may sometimes result in a mismatch between the available vaccine and circulating influenza strains, if the latter have acquired antigenically relevant amino acid substitutions in the HA during this period. The mismatch may increase when mutations occur during the egg-based manufacturing, or when the development process is delayed by one of several recognised potential hurdles (19). Several emerging vaccine technologies, such as mRNA-based vaccines, viral vector systems and nanoparticle-based platforms, are being explored for universal vaccine development (11). **Table 1** provides an overview of several of these cross-protective vaccine technologies in development.

**Table 1:** Strategies/platforms for developing cross-protective vaccines.

Strategy/Platform	Technologies	Mechanism	Examples	Advantages	Challenges
Targeting Conserved Proteins (Multi Epitope-Based Vaccine) (50)	- Heterologous Prime-Boost - Nucleoproteins (NP), or matrix proteins (M2e)-based T-cell vaccines - HA stalk-targeting antibodies	Focuses on highly conserved regions of the virus.	Multimeric-001 (development discontinued) (59)	- Broad protection across strains; - Reduces need for frequent updates.	Limited immunogenicity of conserved regions.
Multivalent Vaccines (51)	- Virus-like particles (VLPs) (67, 68) - Synthetic nanoparticles with HA and NA antigens - Live attenuated influenza vaccines (LAIVs)	Incorporates multiple antigens to increase coverage against diverse influenza strains.	Fluenz^®^ (FluMist) (Nasal live attenuated – EMA & FDA approved) (69, 70)	- Increased stability and immunogenicity compared to subunit vaccines.	Manufacturing complexity and cost.
Delivery Mechanisms	- Viral vector vaccines (e.g., adenovirus-based) (71) - Lipid nanoparticles (e.g., for mRNA)	Improves antigen delivery to cells, enhancing immune response.	VXA-A1.1. (adenovirus vector – universal vaccine in preclinical development) (72, 73)	- Efficiently protects and delivers genetic material; - Stable under standard refrigeration (viral vectors).	Immune interference (viral vectors).
Genetic Encoding (53)	- mRNA vaccines - DNA vaccines	Uses genetic material (mRNA or DNA) to encode antigens for immune system recognition.	mRNA/LNP vaccines (under development) (54, 74)	- Fast development (± 6 weeks); - Scalable production; - Quick to modify for emerging pathogens.	Requires cold chain and storage stability.
Antigen Encapsulation	- Nanoparticle (NP) vaccines (75, 76) - Liposomes	Encapsulates antigens to trigger an immune response.	NanoFlu (under development) (52)	- Enhances antigen stability and delivery; - May be administered intranasally or via aerosols.	- Manufacturing complexity; - Scalability.

FLUniversal partner Vivaldi Biosciences, based in Austria, has developed an alternative vaccine production platform: a Vero cell-based manufacturing system, more flexible compared to egg-based development with a lower risk of changes in the HA protein throughout the process. In addition, the platform used to produce DeltaFLU is readily scalable, with a virtually unlimited substrate – making it preferable to egg-based systems in the event of a pandemic. Technologies improving strain growth and vaccine purity and potency result in a high downstream yield of the influenza vaccine strains lacking NS1. The increased efficiency of the platform amounts to a production time of seven weeks, which is significantly shorter than timelines in conventional influenza vaccine production.

### Preclinical evaluation

Preclinical assessment of DeltaFLU toxicity, safety and tolerability will be performed in ferret models at MediTox S.R.O., based in Czech Republic. After vaccination, ferrets will be extensively assessed for toxicity, including neuro- and immunotoxicity, as well as shedding and biodistribution of the different vaccine strains. Immunology will focus also on nasal IgA levels, and the functional activity of vaccine-induced antibodies against H1N1, H3N2 and influenza B viruses, to demonstrate the universal protection induced by the immunisation strategy. Finally, ferrets will be challenged with highly divergent H1N1, H3N2 and influenza B wild-type strains, in a placebo-controlled setting, to demonstrate protective efficacy of the vaccine.

In addition, immunogenicity of the vaccine will be assessed in a Syrian golden hamster model at the Statens Serum Institute (SSI), based in Denmark. Recent studies highlight that Syrian hamsters are sensitive to influenza viruses, including recent H3N2 strains, without adaptation (20). While hamsters do not resemble human influenza infection as closely as ferrets, they are easier to handle and allow for a wide variety of immunological analyses (21). The hamster model has already been established at SSI using the A/Brisbane/10/07 H3N2 virus, and DeltaFLU will be assessed in the model parallel to the clinical trials. After intranasal vaccination, single-cell-RNA sequencing will be performed on blood, and local secretory IgA responses will be assessed. The vaccinated hamsters will also be challenged with wild-type viruses in a placebo-controlled setting; nasal-associated lymphoid tissue will be evaluated for various cellular responses and correlated with protection against influenza infection. This cutting-edge animal model provides an innovative platform for preclinical vaccine testing.

### Early clinical vaccine evaluation

Following preclinical evaluation, the Centre for Human Drug Research (CHDR) will test the universal DeltaFLU in a first-in-human, phase 1 clinical trial, focusing on safety and immunogenicity for influenza type A group 2. Subsequently, a CHIM study will be conducted in which additional safety data will be collected and protective efficacy will be evaluated in a randomised, placebo-controlled setting. Clinical endpoints (e.g., safety parameters and symptom scores) will be combined with virological and immunological analyses, performed on nasal and blood samples.

For our CHIM, the reverse genetics influenza virus strain A/Texas/71/2017 (H3N2) will be used as a challenge agent. This influenza strain was manufactured according to Good Manufacturing Practice (GMP) in the United States and tested in a dose-titration CHIM at the US National Institute of Allergy and Infectious Diseases (NIAID) to assess safety and infection rate in healthy volunteers (22). An acknowledged issue in the design and execution of CHIMs is the variability in infection rate between continents and study centres, even when identical challenge strains and doses are used. This can be attributed to variability between challenge lots and stability over time, heterogeneity of handling, transport, QC procedures, type and quality of performed assays, sampling techniques and differences in background immunity between regions (23). As a consequence, an important step in the clinical evaluation is to establish a CHIM using the A/Texas/71/2017 strain at the Centre for Human Drug Research (CHDR), based in The Netherlands. In this first implementation trial, 10 healthy volunteers will be inoculated with the challenge strain, without administration of the vaccine. The validation of the challenge agent as well as the harmonisation of clinical procedures (e.g., virus administration, nasal sampling techniques, and virological and immunological analyses) will be the most important focus points of this trial, which is planned to be performed in 2025.

**Figure 1:**
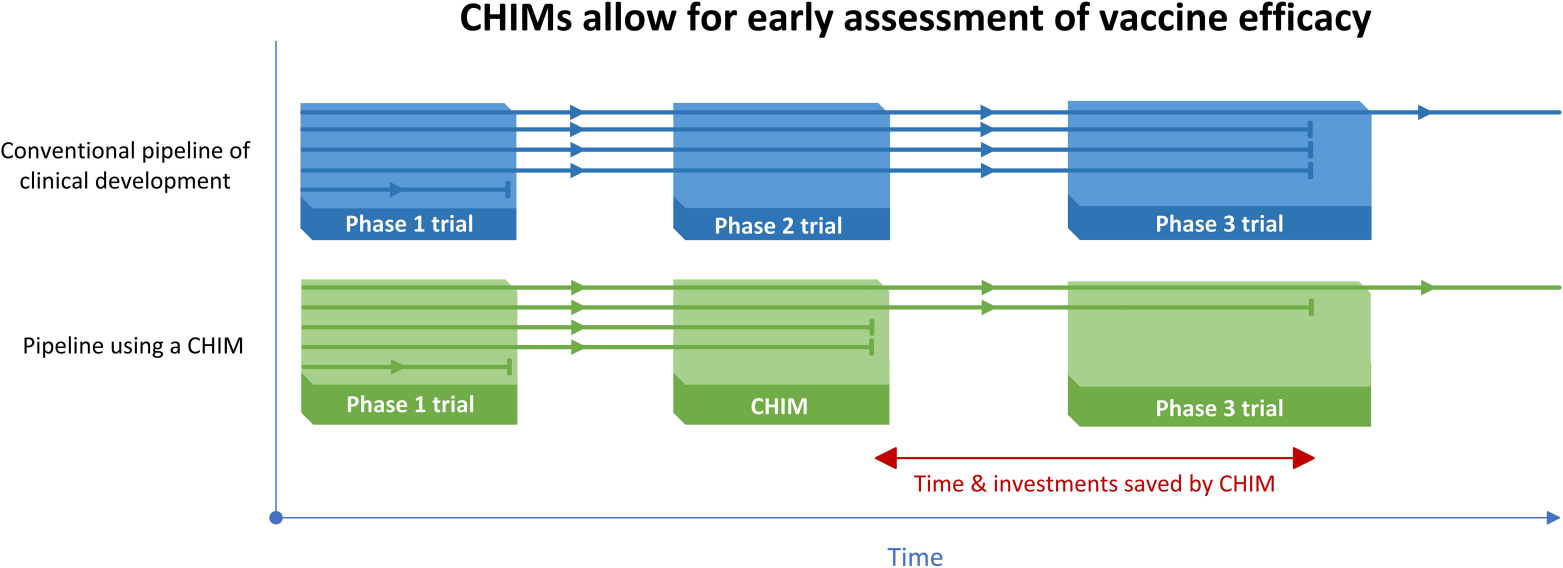
Illustration of the value of using a CHIM with multiple vaccine candidates in the pipeline, with each horizontal line representing a vaccine candidate going through different clinical testing phases. CHIMs allow ineffective vaccines to ‘fail fast’, compared to the conventional vaccine development pathway. This way, resources are saved and pursuing of more promising candidates can be ‘de-risked’ (26).

The principal innovative aspect of a CHIM in the context of vaccine development is the ability to evaluate protective efficacy early in clinical development in a smaller trial compared to conventional phase 2 and 3 studies (24). The WHO endorses the use of influenza CHIMs for vaccine development, as they are generally safe and can provide a wide array of insights (25). While the protective efficacy of a vaccine, as assessed by a CHIM, may not be entirely translatable to a real-world setting, a CHIM can provide the basis for an early go/no-go decision to move a vaccine candidate to the next clinical testing stage. Although CHIMs generally cannot replace conventional real-world efficacy trials, they can nevertheless allow vaccine candidates to ‘fail fast’, i.e., to show a lack of efficacy early in clinical testing, leading to early termination and saving significant time and costs when compared to the conventional pathway of vaccine development. With a pipeline with several vaccine candidates, using a CHIM to gain preliminary information on protective efficacy is highly valuable, as illustrated in **Figure 1**.

The combination of a CHIM implementation study, a conventional phase 1 study, and a placebo-controlled vaccine CHIM study, will provide a comprehensive set of clinical, pharmacological and immunological data on DeltaFLU. Data collected in the influenza CHIM, thoughtfully implemented and validated and with use of harmonised clinical and laboratory procedures, will provide valuable preliminary data on vaccine efficacy, at an early stage in clinical development.

### Immunology and correlates of protection

After the clinical trials assessing DeltaFLU, a substantial collection of clinical samples will be available for immunological analysis. CHIMs can be useful not only for testing of new vaccines and therapeutics, but also to study disease characteristics and to identify immunological factors contributing to and indicative of protection against clinical disease. These correlates of protection (CoPs) are relevant for estimating the protective efficacy of vaccines in an early clinical development stage. CoPs are relatively well established for some pathogens (e.g. serum neutralising antibodies against SARS-CoV-2), while for other pathogens no clear CoP has been established (e.g. respiratory syncytial virus (RSV)) (27, 28). For influenza, a CoP was identified already in 1970s: a virus-specific haemagglutination inhibition (HAI) titre of 1:40 is associated with 50% protection against virus infection, and protection increases as HAI titres rise (29).

Currently, approval of influenza vaccines is based on their ability to increase HAI titres in healthy adults or in older adults; the extensive track record of the HAI assay in trials studying vaccine immunogenicity has eliminated the need for large, costly and time-consuming efficacy trials for a large group of vaccine candidates (12, 30). However, the HAI assay sometimes fails to predict a vaccine’s protective efficacy. There are several aspects of the assay that lead to this limitation: first, the HAI assay is a marker of systemic immunity, providing little information on the local nasal immunity associated with mucosal IgA antibodies (31). Second, it is not representative of cellular immunological processes contributing to protection against infection, which are associated with broader protective immunity and hypothesised to play a significant role in adult immunity against influenza (32). Third, it demonstrates a broad estimate of functional activity of all available HAI-active antibodies and cannot distinguish between antibodies with relatively high or low virus neutralising capacity. Finally, the correlation with protective efficacy is variable or even absent for some vaccine types and technologies, including universal vaccines (9, 33–35).

FLUniversal aims to identify new CoPs for influenza that may circumvent some of these limitations. Using state of the art minimally-invasive approaches to collect upper respiratory tract samples, we will be able to also investigate both humoral (Nasosorptions) and cellular immunity (nasal FLOQswabs) at the site of infection (36–41). These minimally-invasive nasal and blood samples (38), collected during the influenza CHIM, will be analysed using a wide variety of assays: virus neutralising antibodies, HAI antibodies and ELISA antibodies reacting with the whole HA protein, HA subunits and epitopes of the HA stalk; mucosal assays to detect virus neutralising and IgG/IgA antibodies, cytokines and nasal tissue-resident memory T-cells (37). On top of that, spectral flow cytometry will be used to detect and characterise in-depth antigen-specific CD8+ and CD4+ T-cells from nasal samples and PBMCs, whereas memory B-cells (42) will be additionall investigatedin peripheral blood mononuclear cells (PBMCs). Performing the analyses on both blood and nasal mucosa samples allows to understand the effect of compartmentalisation in the context of nasal vaccination. We expect that the combination of mucosal immune measurements with a large number of CHIM samples has the ability to define correlates of protection against influenza infection, which we will then assess also in animal models. FLUniversal aims to use harmonised immunological assays and biological standards to allow comparison within the consortium, and externally (43–45). Immune assays will be performed by the Leiden University Medical Centre (LUMC), The Netherlands; Medicines and Healthcare products Regulatory Agency (MHRA), United Kingdom; and VisMederi, Italy.

The clinical outcomes of the influenza CHIM will be integrated with pre-challenge immunological assay results to identify potential CoPs. Advanced machine learning approaches will be employed to explore relationships between clinical and immunological data, with feature selection performed using Elastic Net regularisation to identify the most predictive immune markers (46, 47) and random forest algorithms (48) to capture complex interactions between immune responses and protection. Transcriptional data will be analysed using mixOmics feature selection (49) to identify key molecular signatures associated with protection. Furthermore, datasets from the clinical study will be compared with protection data from preclinical animal models to establish a comprehensive and robust framework for identifying CoPs.

## Discussion

FLUniversal addresses the critical global health priority of developing new and improved vaccines, particularly universal influenza vaccines. The consortium combines a replication-deficient live attenuated influenza vaccines platform based on deletion of NS1, with innovative technologies for vaccine design, production and immunisation, and state-of-the-art methodologies for preclinical, clinical and immunological evaluation. The progression of DeltaFLU through various stages of vaccine research within a multidisciplinary consortium, integrating complementary areas of expertise, provides significant advantages in enhancing both the effectiveness and efficiency of the vaccine development process.

This initiative is driven by the urgent need for innovative approaches to overcome scientific, logistical, and societal challenges. The need for a universal influenza vaccine is well recognised, and the use of existing vaccine platforms has not yet led to success, shifting the focus towards new technologies as shown in **Table 1** (11). These technologies each face challenges in achieving universal protection: vaccines targeting broadly conserved regions of influenza viruses often require innovative delivery systems or adjuvants to compensate for their poor immunogenicity (50); multivalent vaccines are highly immunogenic and can induce broad protection, yet face challenges in manufacturing scalability and consistency (51, 52); use of genetic platforms such as mRNA and viral vector-based vaccines confer strong but relatively short-lasting immunogenicity, but face limitations in technological requirements for production and storage (53, 54). These novel technologies increasingly gain attention, and combining platforms (e.g., prime-boost regimens targeting conserved T and B cell epitopes), could offer a promising path toward universal or broadly cross-protective protection against influenza (11, 55). Moreover, the definition of a universal influenza vaccine has been topic of discussion: ideally, these vaccines should protect against all influenza A and B viruses, as well as existing or emergent zoonotic viruses with pandemic potential (56). However, it is recognised that this may not be achievable at all, and the WHO has published a Proposed Product Characteristic for universal or broad cross-protective vaccines (57). These discussions, as well as the high failure rate of previous attempts underscore the challenges inherent in vaccine development; an example is the Multimeric-001 vaccine, which failed to show clinical efficacy in a large phase 3 trial, despite robust T-cell responses in earlier trials (58–60). This highlights the importance of early indications of vaccine efficacy, where CHIMs have proven to be particularly advantageous.

CHIMs have emerged as invaluable tools in vaccine research, significantly reducing the risks associated with clinical vaccine testing and potentially avoiding substantial investments in ineffective candidates. Unlike large field trials, which rely on the unpredictable occurrence of natural infections to assess vaccine efficacy, CHIMs provide robust preliminary data on protective efficacy using smaller cohorts and shorter timelines. Historically, CHIMs have successfully accelerated vaccine development for diseases such as cholera, typhoid and malaria, enabling researchers to quickly identify promising candidates and eliminate ineffective ones early on (61–64). CHIMs offer additional advantages: the harmonised timing and collection of samples, coupled with safe and controlled conditions, produce reliable data on virological and immunological processes associated with infection and protection. The establishment of an influenza CHIM at CHDR enables a swift preliminary assessment of DeltaFLU’s protective efficacy; moreover, it provides a comprehensive platform to test future vaccine candidates targeting circulating influenza strains or potential pandemic pathogens. The expertise gained in standardised clinical procedures and harmonised sampling and immunological analyses is adaptable to a wide range of pathogens.

Other international projects focusing on influenza research and vaccines include the Collaborative Influenza Vaccine Innovation Centers (CIVICs) (65) funded by NIAID, and Inno4Vac, funded by the Innovative Medicines Initiative (IMI) (66). Beyond research and development projects, international collaboration is also supported by initiatives such as assay harmonisation—a field where the IMI-funded FLUCOP consortium has made significant contributions (43, 44) – open science, and improved data management. Aligning with these global efforts, FLUniversal aims to promote reproducibility, transparency, and global accessibility of scientific data and methodologies in vaccine development.

In conclusion, the FLUniversal consortium intends not only to develop a universal influenza vaccine, but also to deliver an innovative and versatile vaccine platform, based on novel technologies in vaccine production, as well as an efficient pathway for the (pre)clinical evaluation of vaccine candidates and identification of correlates of protection. This way, FLUniversal can support meeting a critical global health need, contribute to pandemic preparedness and leave a lasting legacy in the form of a versatile and efficient platform for vaccine development.

## Ethics statement

The FLUniversal consortium established an external Ethics Advisory Board (EAB) to provide advice and oversight for the research programs to be carried out by the consortium. The EAB consists of fully independent board members with experience in ethical topics related to the various aspects of the FLUniversal activities. The appointed experts provide the consortium the latest information about ethics and regulations, ensure that the existing rules are adhered to, monitor the work performed by the consortium and advise it when ethics issues arise that are not governed by the ethics routines installed. Potential ethical concerns are reported periodically or *ad hoc* to the EAB. In addition, advice can be sought from the Department of Ethics of FLUniversal partner LUMC, in case this is needed for human studies within FLUniversal. All ethics approvals, measures and considerations will comply with requirements of HCT (for human cells), animal experiments, and authorisations for relevant facilities (i.e., security classification of laboratory, GMO authorisation). A report by the EAB must be submitted at the end of each reporting period of the consortium.

The protocols of the clinical trials to be conducted in the FLUniversal consortium will be submitted for approval by the Dutch Central Committee on Research Involving Human Subjects (CCMO), and the Scientific Advisory Board of CHDR. The studies will be conducted in full compliance with the principles of the Declaration of Helsinki and ICH GCP guidelines. For every participant, written informed consent will be obtained before any study procedures take place. Changes to the protocol regarding trial design and/or safety of participants will only be implemented after approval by the CCMO. FLUniversal aims to involve former challenge trial participants during the writing of the CHIM study protocol(s); either by analysing previously gathered data or by active involvement of initiatives such as 1Day Sooner (https://www.1daysooner.org/). The results of the individual studies will be reported to the Ethics Committee and EAB shortly after the end of the study. In accordance with standard editorial and ethical practice, the results of the studies will be published. Guidelines regarding (co-)authorship, such as the Recommendations for the Conduct, Reporting, Editing, and Publication of Scholarly Work in Medical Journals, will be followed.
